# Equivalent Lung Dose and Systemic Exposure of Budesonide/Formoterol Combination via Easyhaler and Turbuhaler

**DOI:** 10.1089/jamp.2014.1195

**Published:** 2015-12-01

**Authors:** Satu Lähelmä, Ulla Sairanen, Jussi Haikarainen, Jani Korhonen, Mikko Vahteristo, Rainard Fuhr, Merja Kirjavainen

**Affiliations:** ^1^ Orion Corporation Orion Pharma, Espoo, Finland.; ^2^ PAREXEL Early Phase Clinical Unit, Berlin, Germany.

**Keywords:** budesonide, dry powder inhaler, Easyhaler, formoterol, lung deposition, pharmacokinetics, therapeutic equivalence, Turbuhaler

## Abstract

***Background:*** Easyhaler^®^ device-metered dry powder inhaler containing budesonide and formoterol fumarate dihydrate (hereafter formoterol) for the treatment of asthma and chronic obstructive pulmonary disease has been developed. The current approvals of the product in Europe were based on several pharmacokinetic (PK) bioequivalence (BE) studies, and *in vitro-in vivo* correlation (IVIVC) modeling.

***Methods:*** Four PK studies were performed to compare the lung deposition and total systemic exposure of budesonide and formoterol after administration of budesonide/formoterol Easyhaler and the reference product, Symbicort Turbuhaler. The products were administered concomitantly with oral charcoal (lung deposition) and in two of the studies also without charcoal (total systemic exposure). Demonstration of BE for lung deposition (surrogate marker for efficacy) and non-inferiority for systemic exposure (surrogate marker for safety) were considered a proof of therapeutic equivalence. In addition, IVIVC models were constructed to predict study outcomes with different reference product fine particle doses (FPDs).

***Results:*** In the first pivotal study, the exposure and lung dose via Easyhaler were higher compared to the reference product (mean comparison estimates between 1.07 and 1.28) as the FPDs of the reference product batch were low. In the following studies, reference product batches with higher FPDs were utilized. In the second pivotal study, non-inferiority of Easyhaler compared to Turbuhaler was shown in safety and BE in efficacy for all other parameters except the formoterol AUC_t_. In the fourth study where two reference batches were compared to each other and Easyhaler, budesonide/formoterol Easyhaler was bioequivalent with one reference batch but not with the other having the highest FPDs amongst the 28 reference batches studied. In the IVIVC based study outcome predictions, the test product was bioequivalent with great proportion of the reference batches. For the test product and the median FPD reference product BE was predicted.

***Conclusions:*** Equivalence regarding both safety and efficacy between budesonide/formoterol Easyhaler and Symbicort Turbuhaler was shown based on totality of evidence from the PK studies and IVIVC analyses, and therefore, therapeutic equivalence between the products can be concluded. The results of the PK studies are likely dependent on the variability of FPDs of the reference product batches.

## Introduction

Asthma and chronic obstructive pulmonary disease (COPD) represent inflammatory airway diseases that cause significant health problems to patients and a substantial economic burden on societies.^(1,2)^

During the last decades, inhaled corticosteroids (ICS) have been the first-line treatment for patients with persistent asthma irrespective of disease severity.^(1,3,4)^ Based on treatment guidelines, patients with asthma not sufficiently well controlled with ICS alone (plus a rapid-acting bronchodilator used as needed) should have a long-acting β_2_-agonist (LABA) added.^(1)^ This combination therapy has an obvious scientific rationale as LABA and ICS may optimize each other's beneficial actions in the airways.^(5)^ Combining these two medications in one inhaler may simplify the dosing regimen and improve adherence to prescribed therapies for patients for whom combination therapy is appropriate.^(6,7)^ Fixed combination inhalers (dry powder inhalers, DPIs, and pressurized metered dose inhalers, pMDIs) containing both an ICS and a LABA (e.g., budesonide/formoterol fumarate dihydrate (hereafter formoterol), fluticasone propionate/salmeterol, or fluticasone propionate/formoterol) currently have an established position among the treatment options of asthma.

The originator budesonide/formoterol combination, Symbicort^®^ Turbuhaler (AstraZeneca, London, United Kingdom) was initially used only as maintenance treatment with one or two administrations daily, but an adjustable maintenance therapy was subsequently developed.^(8,9)^ Later, a posology was accepted by regulatory authorities with maintenance therapy plus additional doses as needed (called SMART; Symbicort Maintenance And Reliever Therapy).^(10)^ Several asthma studies have shown the clinical advantage of the budesonide/formoterol SMART therapy.^(11)^ The safety profile of budesonide/formoterol has also been thoroughly documented.^(12)^ The use of a combination inhaler incorporating both ICS and LABA in patients with asthma ensures that, as stated in the GINA guideline, the LABA is not administered alone.^(1)^ In patients with COPD the ICS/LABA combinations have been shown to improve airway function, reduce symptoms, improve quality of life, prevent exacerbations, and prolong the time to the next exacerbation.^(13,14)^ Comprehensive reviews of the use of the budesonide/formoterol in patients with COPD have also been published.^(15,16)^

Orion Pharma (Espoo, Finland) has developed a budesonide/formoterol combination to be delivered via the Easyhaler^®^, a device-metered DPI. The mono-components of the product, budesonide and formoterol, as well as salbutamol and beclometasone, are available on the market in the Easyhaler inhaler. Addition of the ICS/LABA combination to the Easyhaler product portfolio was considered important because patients may benefit from use of only one type of inhaler for their medication.^(17)^ In the development of the product, the European Medicine Agency (EMA) guideline on the requirements for clinical documentation for orally inhaled products (OIPs)^(18)^ has been followed. According to the guideline, a second entry orally inhaled combination product has to demonstrate therapeutic equivalence with the reference combination product for both active substances of the test combination product. In case therapeutic equivalence cannot be proven based on *in vitro* data, pharmacokinetic (PK) and clinical studies are required. We report here the results of four PK studies that evaluated whether equivalent pulmonary deposition (lung dose after blocking of the gastro-intestinal, GI, uptake with charcoal) and systemic exposure (without charcoal blockage) were demonstrable after inhalation of budesonide/formoterol via Easyhaler and Turbuhaler.

## Materials and Methods

### Study drugs

Symbicort Turbuhaler Forte (320 μg budesonide/9 μg formoterol per inhalation) as the reference product (hereafter Symbicort Turbuhaler) and budesonide/formoterol Easyhaler 320/9 μg per inhalation were the investigational medicinal products in all four studies. In addition, respective placebos were needed in double-blind studies (double-dummy approach), and for charcoal block Carbomix granules (Leiras Takeda, Helsinki, Finland) were utilized.

### Study subjects

Healthy male and female subjects aged 18–60 years with a body mass index (BMI)>19 and<30 kg/m^2^, weight at least 50 kg, a forced expiratory volume in one second (FEV_1_)≥80% of predicted normal, and good general health were recruited for the studies. Smokers of more than five cigarettes per day were excluded, as were pregnant or breast-feeding females and those of childbearing potential not using adequate contraception.

### Methods

**FIG. 1.**
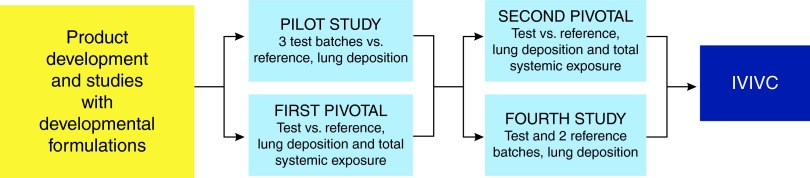
Flow chart of budesonide/formoterol Easyhaler pharmacokinetic studies. IVIVC, *In vitro-in vivo* correlation.

This report consists of four PK studies on inhaled budesonide/formoterol administered by Easyhaler and Turbuhaler. The flow of the studies is shown in Figure 1. The pilot and the first pivotal study were performed in parallel, and after them the second pivotal study and the fourth study, also in parallel. In the pilot study, lung deposition of budesonide and formoterol was assessed and compared after administration of three different batches of budesonide/formoterol Easyhaler, and one Symbicort Turbuhaler batch. The aim of the pivotal studies was to demonstrate both BE in terms of lung deposition and non-inferiority in terms of systemic exposure between the products. The primary aim of the fourth study was to evaluate the acceptance range with which two Symbicort Turbuhaler batches (A and B) could be declared bioequivalent (BE). The secondary objective of the study was to compare PK parameters of budesonide/formoterol Easyhaler with the Turbuhaler batches.

In all studies the same budesonide/formoterol Easyhaler batch was included (in the pilot also two additional Easyhaler batches). For the reference product four different batches were utilized. The same reference product batch was used in the first two studies. In the second pivotal study one and in the fourth study two new reference product batches were introduced.

Reference product fine particle doses (FPDs, the mass of particles under 5 μm) were studied throughout the budesonide/formoterol Easyhaler development program with increasing number of batches over time. Altogether six batches had been purchased and analyzed before the start of the first two studies, whereas the number of batches was 24 before the start of the last two studies. Four additional batches were purchased during the registration process of the product, resulting altogether in 28 reference product batches in the database. FPDs were determined according to the *in vitro* testing of DPIs established by the European Pharmacopoeia monograph Preparations for Inhalation^(19)^ using Next Generation Impactor (NGI, apparatus E). The number of inhalers analyzed was typically between three to five for both Easyhaler and Turbuhaler. The *in vitro* studies were performed by Oy Medfiles Ltd, Kuopio, Finland.

The PK studies were performed according to a 3 or 4-period, 3 or 4-treatment, crossover design. The pilot study was an open study, but all others were carried out as double-blind with double-dummy technique. Study treatments, a single dose consisting of two inhalations of the budesonide/formoterol 320/9 μg per inhalation via Easyhaler or Turbuhaler (total dose 640/18 μg), were administered in a randomized order concomitantly with charcoal in all studies and in the pivotal studies also without charcoal.

The charcoal regimen used to block the GI absorption was as follows: immediately before study treatment administration, the mouth was thoroughly rinsed with 50 mL of charcoal suspension before swallowing. The charcoal administration was repeated immediately after study treatment and again when 45 min and 1 h 30 min had elapsed. The efficiency of the block was confirmed in a separate PK study in healthy volunteers. A single oral dose of 640/18 μg of budesonide/formoterol was administered with and without the charcoal and the blockage of GI absorption was found to be 98.8% for budesonide and 99.8% for formoterol (data on file).

In all studies the subjects were trained in the correct use of the inhalers at the screening visit and before each study drug administration. The studies consisted of a screening period, three or four treatment days separated by at least 3-day wash-out periods, and an end-of-study visit occurring at least 3 days after the last study treatment administration. Blood samples for the determination of budesonide and formoterol concentrations in plasma were drawn before the administration of the study treatments and up to 12 h for budesonide and up to 24 h for formoterol after drug administration. The sampling time points (hours:minutes) after the administration of the study drugs were: 0:05, 0:07, 0:10, 0:15, 0:20, 0:30, 0:45, 1:00, 1:30, 2:30, 4:00, 6:00, 8:00, and 12:00 for both drugs, and in addition 24:00 for formoterol analysis only. Budesonide and formoterol concentrations in plasma were determined by separate, validated liquid chromatography-tandem mass spectrometry (LC-MS/MS) methods at PPD, Madison, WI, USA. The lower limit of quantification (LLOQ) for the budesonide method P4290.01^(20)^ was 10 pg/mL and for the formoterol method P860.02^(21)^ 0.5 pg/mL. The bioanalytical analyses were performed according to the principles of applicable good laboratory practice (GLP) and good clinical practice (GCP).

As primary markers of efficacy, the following variables were calculated from concentration-time curves for budesonide and formoterol after study drug administration with charcoal: the maximum observed concentration of concentration-time curve (C_max_) and the area under the concentration-time curve from time zero to the last sample with quantifiable drug concentration (AUC_t_) calculated with the linear trapezoidal rule. The secondary PK parameters were the area under the concentration-time curve from time zero to infinity (AUC_∞_) determined by adding AUC_t_ to the extrapolated area that was determined dividing the last quantifiable concentration by λ_z_ (λ_z_=the terminal elimination rate constant from log-linear portion of a concentration-time curve), the time to reach the maximum concentration (t_max_), and the terminal elimination half-life (t_½_) calculated with the equation ln2/λ_z_. As surrogate markers for safety, the same PK variables as above were calculated after administration of the test and the reference products without GI charcoal block. The PK parameters were calculated by a noncompartmental method using the WinNonlin^®^ 5.0.1 (Certara L.P, St. Louis, MO, USA) computer program. The actual time of sampling was used in the calculations. The zero time was the start of the first inhalation of the active study treatment.

Clinical safety was assessed by supine heart rate (HR), systolic and diastolic blood pressure (BP), 12-lead electrocardiogram (ECG), physical examination, laboratory safety assessments, and adverse events (AEs). The pilot and the fourth study were performed at PAREXEL Early Phase Clinical Unit, Berlin, Germany, and the pivotal studies at Orion Pharma Clinical Pharmacology Unit, Espoo, Finland. All four studies were performed according to GCP and the Declaration of Helsinki. The study protocols were approved by the national regulatory authorities and ethics committees before the start of the study procedures. All subjects gave their written informed consent to participate in the studies.

### Statistical methods

The determination of sample size for individual studies was based on previous studies with the developmental formulations of budesonide/formoterol Easyhaler product. The mean squared error (MSE) of budesonide C_max_ was the highest of the primary parameters and was therefore used in the sample size calculations. It was assumed that the expected ratio of means would be 0.9–1.1. The per protocol (PP) data set was used when comparing the PK results. The PP data set excluded all the subjects who discontinued, had a major protocol deviation, or insufficient number of PK samples for the calculation of reliable PK parameters. The primary PK variables for lung deposition, C_max_ and AUC_t_, were analyzed using a general linear mixed model. The responses were modeled using logarithmic transformations. By taking exponential back-transformations, the results were returned to the original scale, yielding the ratio of geometric means and their 90% confidence intervals (CIs). These CIs were evaluated against the conventional BE region from 0.80 to 1.25. The secondary PK variables were AUC_∞_, t_max_ and t_½_. AUC_∞_ was analyzed in the same way as C_max_ and AUC_t_. The primary safety variables C_max_ and AUC_t_ (administration without charcoal) were analyzed and described as above for BE. Non-inferiority (i.e., not having higher exposure after test than after the reference product) for both budesonide and formoterol was evaluated. The upper limit of the one-sided 95% CI for the ratio of the geometric means of primary PK parameters was not to exceed 1.25.

### *In vitro–in vivo* correlation *(IVIVC)*

The lung deposition data obtained was further explored from an IVIVC perspective by constructing models to predict study outcomes with different reference product FPDs. The T/R-ratios of the primary parameters (AUC_t_ and C_max)_ versus the T/R-ratios of FPDs of the batches under comparison were used. A linear regression was built separately for all primary PK parameter T/R-ratios to model them with the FPD ratios. The modeling was done based on altogether five comparisons between the Easyhaler batch and four reference batches (i.e., with all comparisons available after administration of the products concomitantly with charcoal). Validation of the predictability of the models was carried out as instructed in the regulatory guidance.^(22,23)^ All the reference batches with FPDs falling within the limits of±15% of the median FPD were used in the predictions (*n*=26). A prediction for the comparison between budesonide/formoterol Easyhaler and the median FPD reference product batch was also carried out.

All statistical analyses were performed with SAS^®^ for Windows (SAS Institute Inc., Cary, NC, USA).

## Results

**FIG. 2.**
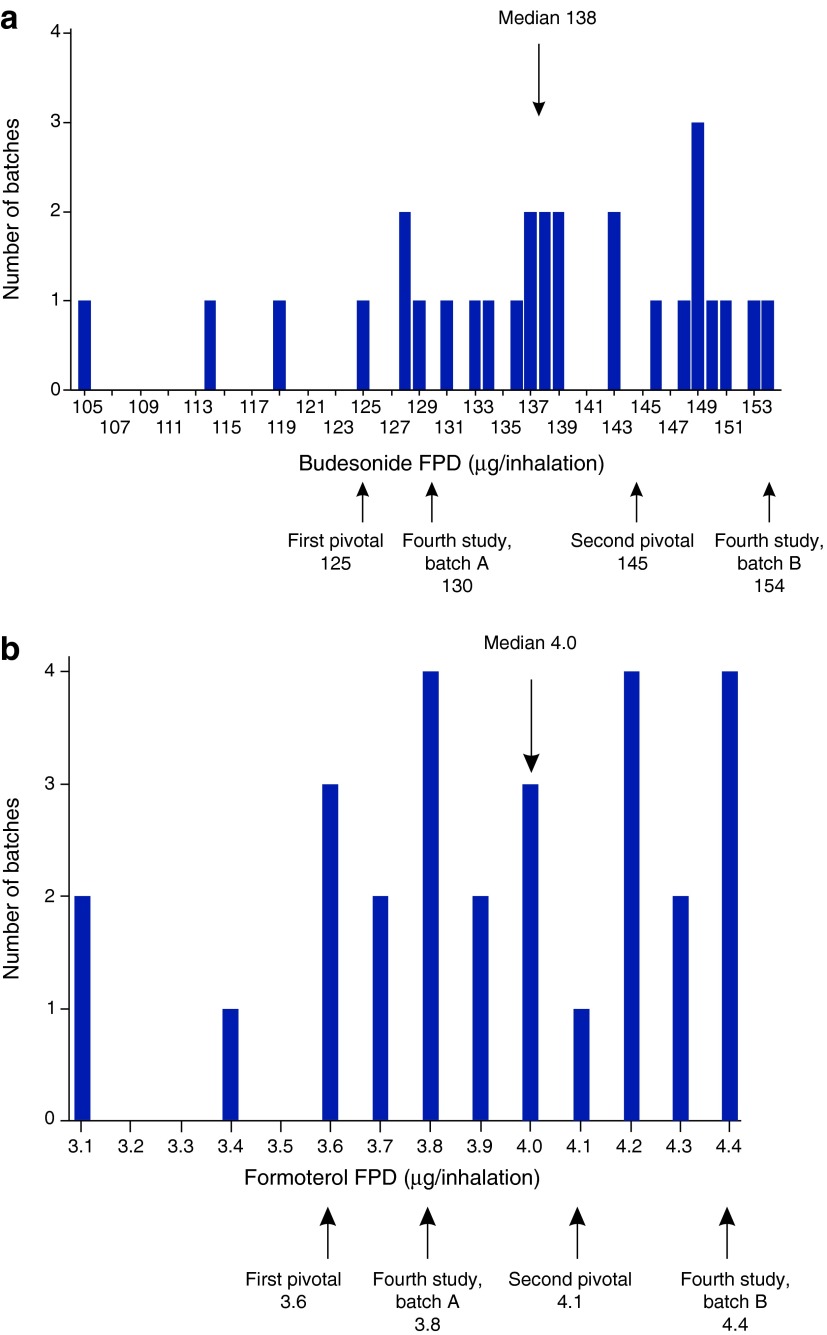
Budesonide **(a)** and formoterol **(b)** fine particle doses (FPDs, μg/inhalation) of the tested reference product batches (*N*=28).

There is notable batch-to-batch variability in the FPDs of the reference product (Fig. 2). The mean FPDs of the batches utilized in the PK studies varied between 125 and 154 μg/inhalation for budesonide and between 3.6 and 4.4 μg/inhalation for formoterol. The median FPD was 138 μg/inhalation for budesonide and 4.0 μg/inhalation for formoterol.

There were no major differences in demographic and baseline characteristics of the subjects in the studies (Table 1).

**Table 1.** Demographic Data (ITT Population)

	*Pilot study*	*First pivotal*	*Second pivotal*	*Fourth study*
No. of subjects	17	74	72	48
Females, %	47	50	53	48
Mean age (range) years	37 (22–51)	31 (18–59)	27 (18–57)	44 (18–55)
Mean weight (range) kg	74 (51–95)	71 (50–111)	70 (52–105)	77 (57–103)
Mean height (range) cm	173 (154–188)	174 (156–198)	174 (159–197)	174 (156–188)
Mean BMI (range) kg/m^2^	25 (20–30)	23 (19–30)	23 (19–30)	25 (21–30)
Mean FEV_1_, % of predicted (range)	109 (83–128)	98 (80–128)	98 (81–126)	109 (87–147)

**FIG. 3.**
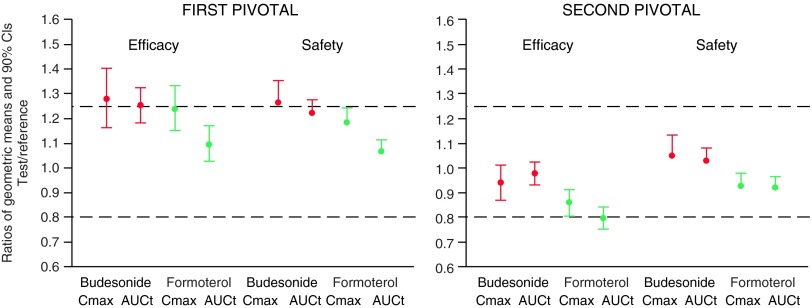
Summary of budesonide and formoterol C_max_ and AUC_t_ comparisons between the test and the reference product in the pivotal studies (PP population). The bioequivalence/noninferiority limits are shown with *dotted lines*.

In the first pivotal study, absorption of both budesonide and formoterol was slightly higher from budesonide/formoterol Easyhaler than from Symbicort Turbuhaler after administration with (*N*=69) and without (*N*=65) charcoal (Tables 2 and 3, Fig. 3). Three out of the eight primary parameters fulfilled the pre-specified BE/non-inferiority criteria, but for the rest of the parameters the results were inconclusive with CIs for the T/R-ratios overlapping the acceptance range. In regard to T/R comparisons, the results of the pilot study (*N*=16) were in line with the results of the first pivotal study. Differences in PK parameters between the different Easyhaler batches (A, B, and C) were small (Tables 2 and 3).

**Table 2.** Primary Pharmacokinetic Parameters of Budesonide After Single Dose of Two Inhalations of Budesonide/Formoterol Easyhaler 320/9 μg/Inhalation and Symbicort Turbuhaler Forte (PP Population)

	*Budesonide C_max_ (pg/mL)*		*Budesonide AUC_t_ (h×pg/mL)*	
	*Mean (90% CI)*	*Intra-subject CV (%)*	*Mean (90% CI)*	*Intra-subject CV (%)*
*Pilot*				
B/F Easyhaler (batch A)	2030 (1744–2363)	22	4494 (4001–5048)	17
B/F Easyhaler (batch B)	1881 (1616–2190)		4577 (4075–5141)	
B/F Easyhaler (batch C)	1841 (1582–2143)		4955 (4411–5565)	
Symbicort Turbuhaler	1506 (1294–1753)		3677 (3274–4130)	
*First pivotal*				
B/F Easyhaler	1978 (1832–2135)	34	4867 (4618–5129)	20
Symbicort Turbuhaler	1543 (1430–1666)		3881 (3683–4090)	
B/F Easyhaler without CC	2138 (1990–2298)	30	5403 (5144–5674)	15
Symbicort Turbuhaler without CC	1690 (1572–1816)		4415 (4204–4637)	
*Second pivotal*				
B/F Easyhaler	1709 (1599–1826)	28	4492 (4296–4697)	17
Symbicort Turbuhaler	1816 (1699–1940)		4590 (4389–4799)	
B/F Easyhaler without CC	1966 (1824–2119)	28	5103 (4845–5376)	16
Symbicort Turbuhaler without CC	1875 (1739–2021)		4937 (4687–5200)	
*Fourth study*				
B/F Easyhaler	1823 (1661–2001)	28	4144 (3878–4428)	20
Symbicort Turbuhaler (batch A)	1730 (1576–1899)		3787 (3544–4047)	
Symbicort Turbuhaler (batch B)	1996 (1818–2190)		4242 (3970–4533)	

B/F Easyhaler, budesonide/formoterol Easyhaler; CV%, coefficient of variation; mean, estimated geometric mean; 90% CI, 90% confidence interval for the mean. Administration with concomitant charcoal except when separately mentioned (without CC).

**Table 3.** Primary Pharmacokinetic Parameters of Formoterol After Single Dose of Two Inhalations of Budesonide/Formoterol Easyhaler 320/9 μg/Inhalation and Symbicort Turbuhaler Forte (PP Population)

	*Formoterol C_max_ (pg/mL)*		*Formoterol AUC_t_ (h×pg/mL)*	
	*Mean (90% CI)*	*Intra-subject CV (%)*	*Mean (90% CI)*	*Intra-subject CV (%)*
*Pilot*				
B/F Easyhaler (batch A)	33 (28–39)	24	63 (55–73)	21
B/F Easyhaler (batch B)	34 (29–40)		71 (62–82)	
B/F Easyhaler (batch C)	35 (30–41)		71 (61–81)	
Symbicort Turbuhaler	29 (25–34)		64 (55–74)	
*First pivotal*				
B/F Easyhaler	40 (37–43)	26	86 (81–91)	23
Symbicort Turbuhaler	32 (30–34)		78 (73–83)	
B/F Easyhaler without CC	41 (38–44)	21	106 (101–112)	15
Symbicort Turbuhaler without CC	35 (32–37)		99 (94–105)	
*Second pivotal*				
B/F Easyhaler	33 (31–35)	23	74 (71–78)	19
Symbicort Turbuhaler	38 (36–40)		93 (88–98)	
B/F Easyhaler without CC	35 (33–37)	19	97 (92–102)	16
Symbicort Turbuhaler without CC	38 (36–40)		105 (100–110)	
*Fourth study*				
B/F Easyhaler	21 (19–23)	26	42 (38–47)	31
Symbicort Turbuhaler (batch A)	23 (21–26)		47 (42–52)	
Symbicort Turbuhaler (batch B)	27 (25–30)		59 (53–66)	

B/F Easyhaler, budesonide/formoterol Easyhaler; CV%, coefficient of variation; mean, estimated geometric mean; 90% CI, the 90% confidence interval for the mean. Administration with concomitant charcoal except when separately mentioned (without CC).

**FIG. 4.**
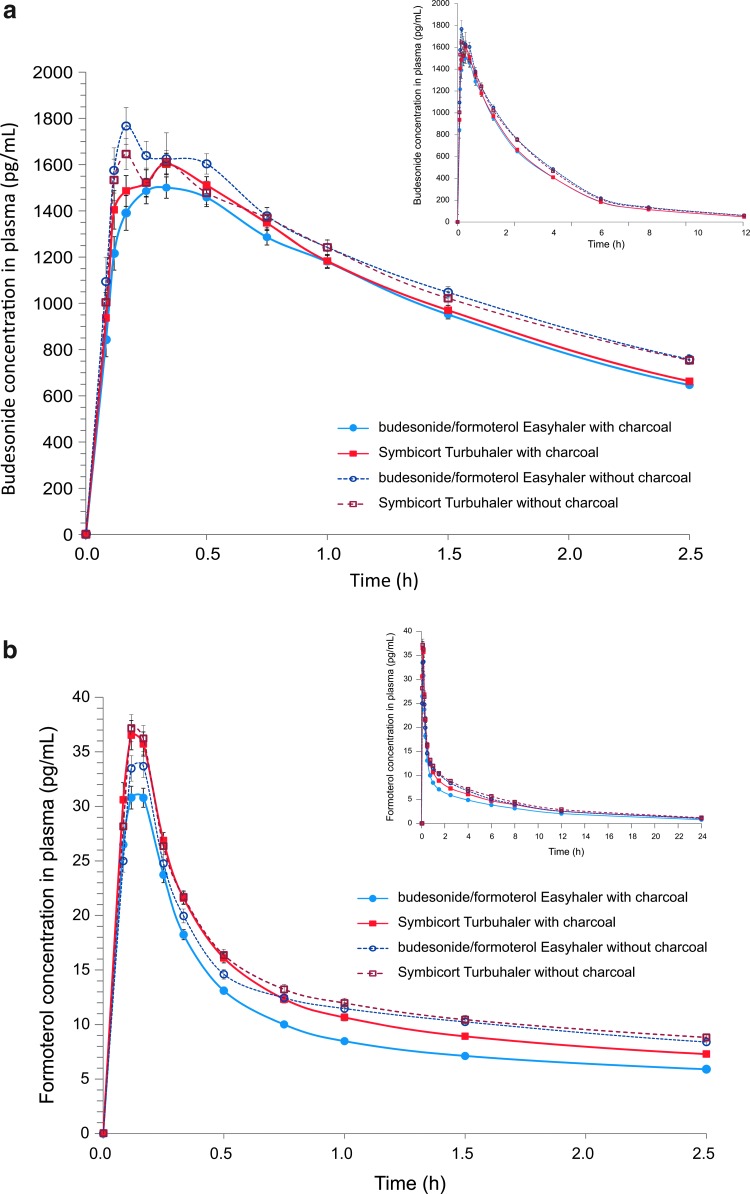
Budesonide **(a)** and formoterol **(b)** concentrations in plasma (pg/mL) after two inhalations of budesonide/formoterol Easyhaler 320/9 μg/inhalation and Symbicort Turbuhaler Forte with and without concomitant charcoal administration in the second pivotal study (PP population, mean±SE).

As the FPDs of the reference product studied in the first two studies were found to be relatively low (Fig. 2) two additional studies were carried out (Fig. 1). In the second pivotal study (*N*=69 for lung deposition and *N*=65 for total systemic exposure comparisons) all except one of the eight primary parameters fulfilled the pre-specified BE and non-inferiority criteria (Fig. 3). However, the CI of formoterol AUC_t_ exceeded (but overlapped) the acceptance limit of 0.80 (contrary to the first pivotal study in which the overlap was at the upper limit). In other words, the first pivotal study results showed higher absorption after inhalation via Easyhaler (comparison estimates>1), whereas in the second pivotal the estimates were closer to 1 and within the acceptance limits except for formoterol AUC_t_. The shapes of the budesonide and formoterol plasma concentration mean curves were similar for both products, showing similar absorption and elimination profiles in general (Fig. 4).

**FIG. 5.**
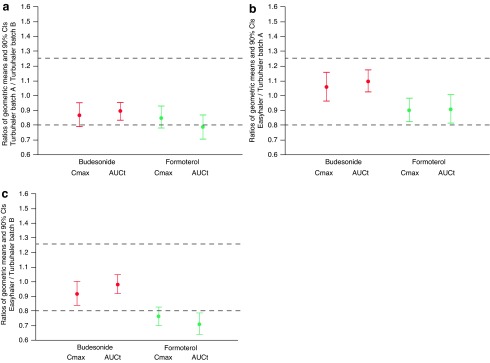
Summary of budesonide and formoterol C_max_ and AUC_t_ comparisons between Symbicort Turbuhaler batches A and B **(a)**, between budesonide/formoterol Easyhaler and Symbicort Turbuhaler batch A **(b)**, and between budesonide/formoterol Easyhaler and Symbicort Turbuhaler batch B **(c)** in the fourth study (PP population). The bioequivalence limits are shown with *dotted lines*.

The results of the fourth study, which utilized two Symbicort Turbuhaler batches (A and B), are shown in Figure 5. For the PK parameter comparisons (*N*=47–48), BE between the two reference batches could not be demonstrated. The comparison of budesonide/formoterol Easyhaler with Symbicort Turbuhaler batch A showed BE with geometric means and 90% CIs between 0.8 and 1.25. However, when comparing budesonide/formoterol Easyhaler with Symbicort Turbuhaler batch B BE was not shown as a reflection of high FPDs of Turbuhaler batch B.

The secondary parameters t_max_ and t_½_ were comparable between the test and the reference products in all studies. The median budesonide t_max_ varied from 10 to 20 minutes and median formoterol t_max_ from 5 to 8 minutes in different studies irrespective of the product. Mean t_½_ for budesonide varied from 3.1 to 3.6 h and mean t_½_ for formoterol from 8.5 to 12 hours. The obtained AUC_∞_ values were in line with the corresponding AUC_t_ values.

**FIG. 6.**
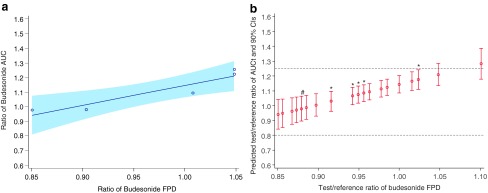
IVIVC model for budesonide AUC_t_ **(a)**, and prediction of T/R-ratio of budesonide AUC_t_ with different FPD ratios (T/R-ratio estimates and 90% CIs, 26 different reference batches, *2 batches, #3 batches) **(b)**.

**FIG. 7.**
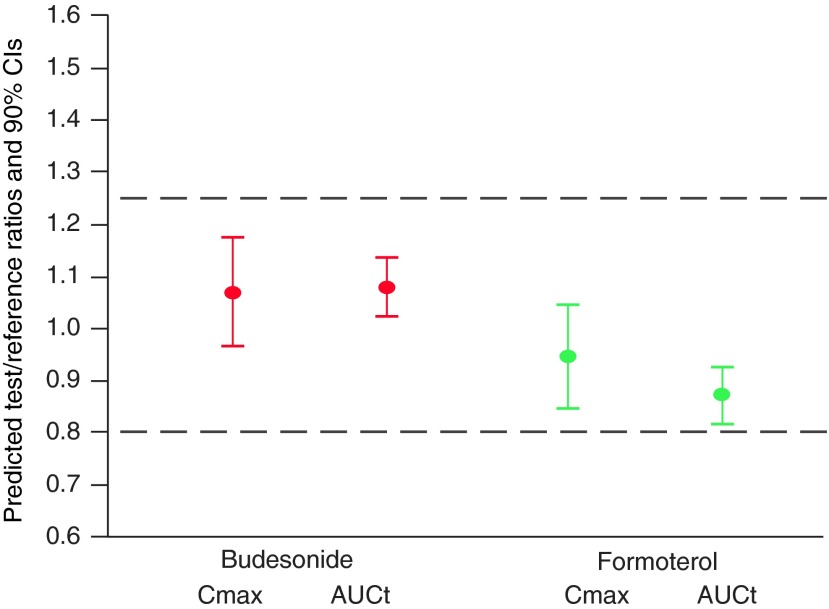
Predicted study outcome for budesonide/formoterol Easyhaler and the median FPD reference product batch comparison (estimated T/R ratios and 90% CIs).

Based on the results there was a rank order correlation between FPDs of the reference product and the PK results obtained (i.e., when a reference product had low FPDs it resulted in high T/R-ratios of PK parameters and vice versa). Therefore, IVIVC models were constructed for the primary parameters. As an example of the IVIVC models, the model for budesonide AUC_t_ and the respective predictions for different reference product batches are shown in Figure 6. Based on the results, the test product is bioequivalent with great proportion of the reference batches. The equation for the model is y=−0.22+1.36x, where y is the T/R-ratio of budesonide AUC_t_ and x is T/R-ratio of budesonide FPD. The IVIVC models had high coefficients of determination (between 0.77 and 0.88), indicating that a large proportion of the variation in the T/R-ratio of PK parameters could be explained by the variation in the FPD of the reference product. In the validation of the predictability, all the average absolute percent prediction errors were≤10% and all the individual absolute percent prediction errors were≤15%, suggesting a good predictive performance by the models. In addition to the predictions of the study outcomes with different reference batches, a prediction for the comparison between budesonide/formoterol Easyhaler and a reference product batch with median FPDs was carried out. The prediction shows bioequivalent lung deposition between the test and reference product (Fig. 7).

There were no safety issues in any of the studies and no serious AEs were reported. AE profiles were similar after both inhalers. Altogether 3 subjects discontinued due to an AE. One subject discontinued in the first pivotal study due to vasovagal syncope after Easyhaler administration, and one subject in the second pivotal study due to tremor of the whole body after Turbuhaler. Both events were assessed as related to the study treatment. In the pilot study, one subject discontinued due to vasovagal reaction (presyncope) due to blood sampling. The events resolved spontaneously.

## Discussion

In support of efficacy and safety of budesonide/formoterol Easyhaler, PK studies were performed to compare the lung dose and total systemic exposure of budesonide and formoterol with the reference product. An open pilot study and three double-blind, randomized, crossover, single dose studies were carried out. In the pilot and the first pivotal studies, the exposure and lung dose of budesonide and formoterol via Easyhaler were higher compared to the reference product. In these studies the FPDs of the reference product batch were fairly low. In the following pivotal and the fourth study, different reference product batches with higher FPDs were utilized. In the second pivotal study, non-inferiority of Easyhaler compared to Turbuhaler was shown in safety and equivalence in efficacy was demonstrated for all other parameters except the formoterol AUC_t_. In the fourth study budesonide/formoterol Easyhaler was bioequivalent with one Symbicort Turbuhaler batch but not with the other, having the highest FPDs amongst the 28 batches studied.

Because budesonide/formoterol Easyhaler batch was the same in all studies, the study results suggest that a reference product with high FPDs results in lower test/reference–ratios of the PK parameters and a reference product with low FPDs results in higher ratios. To further evaluate the relationship between the PK parameters and *in vitro* measured FPDs, IVIVC models were constructed. The predicted study outcome results covered the BE acceptance range (0.80–1.25) from the low to the high end even when the reference product FPDs were within±15% of the median. For the test product and the median FPD reference product bioequivalent lung dose was predicted.

The EMA guideline on the requirements for clinical documentation for OIPs has been followed in the development of budesonide/formoterol Easyhaler to the extent possible and necessary. The guideline provides a stepwise approach to demonstrate therapeutic equivalence between inhaled products, the originator, and a second entry product. The first step involves *in vitro* comparisons between the test and reference products. In some cases, the use of only comparative *in vitro* data may be considered acceptable if the product satisfies all the criteria set out in the guideline. *In vitro* comparisons did not show complete equivalence between budesonide/formoterol Easyhaler and Symbicort Turbuhaler. This is typical for DPIs not resembling the originator in design. Hence, PK studies (second step) in healthy volunteers were performed. The data from the studies together with the required *in vitro* investigations form the basis of budesonide/formoterol Easyhaler marketing authorizations in Europe.^(24)^

The guideline calls for the use of intended patient population in PK trials with OIPs.^(18)^ The use of healthy volunteers deviates from this principle but is currently accepted by the regulatory authorities as healthy volunteers are considered less variable and more discriminative than patients with asthma.^(25)^ The findings of studies in healthy volunteers can be bridged to patients when the flow rate dependency characteristics of the products can be considered similar. Budesonide/formoterol Easyhaler and Symbicort Turbuhaler display similar patterns of flow rate dependency within clinically relevant flow limits in asthma and COPD patients.^(26)^

As an option to PK studies, lung dose of an inhaled drug can be assessed by using an imaging study.^(18)^ Imaging studies might give a better estimate especially on regional quantification of the pulmonary deposition compared to PK studies but there are challenges related to their performance (e.g., validation of radiolabeling of drug formulation, short half-life of some radionuclides) and standardization of methodology.^(27)^ At present, the European authorities consider plasma concentrations obtained in a PK study to be indicative of the concentrations at the site of action,^(28)^ and the development programs of the most recently approved products have employed PK studies rather than imaging studies when comparing lung dose between an originator and a second entry product. ^(24,25,29)^

The objective of developing an IVIVC in general is to establish a predictive mathematical model describing the relationship between an *in vitro* property and a relevant *in vivo* response. The number of publications on IVIVC for OIPs is limited. It has been suggested that there would be a relationship between the *in vitro* respirable dose and the relative amount delivered to lungs measured using PK methods or gamma scintigraphy^(30,31)^ and there is previous evidence of the FPD being a good predictor for lung bioavailability.^(32,33)^ The current regulatory guidance for IVIVC is only applicable to oral dosage forms.^(22,23)^ Even though the PK studies conducted were similar in the respect of the study design and study subjects the limitation of the IVIVC is that the studies were not planned for IVIVC development purposes but models were constructed retrospectively. The other limitation of the models is the lack of different test product batches. However, the Easyhaler batch studied was found to be representative among the manufactured production scale batches.

The practical challenges in performing PK studies with OIPs are many. A number of details need to be standardized but nevertheless mistakes can occur. The correct inhalation technique for the devices may differ resulting in inhalation bias. Therefore, the inhalation technique for both devices was taught and practiced beforehand. The aim was to minimize variability caused by subject-related factors, of which inhalation technique was considered to be the most critical. The manufacturers' instructions were used. The subjects adopted the techniques well and the variability of the PK parameters was of similar magnitude as in a previous study with Turbuhaler in healthy volunteers.^(34)^ In a double-blind trial study, personnel remains objective and if unsuccessful administration occurred the study period could be discontinued before blood sampling and repeat visit organized. Re-scheduling periods was limited to maximum of two per subject in our studies. However, only eight periods in total were re-scheduled (approximately 1%). Intense blood sampling was also well managed by the experienced study personnel. For drug substances with early t_max_ like formoterol frequent sampling shortly, a few minutes, after dosing is essential.

During the development, the reference product was extensively studied *in vitro* to obtain a solid understanding of reference product characteristics. The results of the reference product FPD analysis (28 batches) confirmed that there is batch to batch variability in the FPDs. This is typical for the dosage form in question and understandable in the light of *in vitro* specifications of approved OIPs which allow FPD variance of ±20% to ±45% of the mean.^(35)^ The overall number of studied reference product batches can be considered high, bearing in mind that the availability of different batches on the market at certain time is limited. Due to a limited availability, procurement may need to be spread over fairly long period, as in our case over approximately a year and a half. Therefore, it is believed that the gathered database is a good illustration of the *in vitro* performance of the reference product and a strong basis for a reliable batch to batch comparison. Naturally the FPD result level may be a subject to the measurement set-up specific to the laboratory in question unless the exact methods have been established and validated elsewhere. However, regardless of the result level the use of the same set-up reveals the differences between the batches. All FPD analyses were carried out by the same external laboratory.

In the fourth study the test product's performance in relation to close to median FPD reference batch and an extreme FPD batch (high) was demonstrated. Lung dose after Easyhaler was bioequivalent with the former but not with the latter. Based on the study results, it also appears evident that two reference product batches on the market with different FPDs might not be bioequivalent when tested in a sensitive PK study setting. However, the results have to be considered also against the extensive published clinical data with the reference product for the treatment of asthma and COPD.^(8–11)^

Even though BE was not shown for lung deposition between the batches, there are no data available showing that efficacy or safety of the product is compromised from batch to batch (within the approved specification limits). This would suggest the high discriminative nature of PK studies over clinical studies. Studies by Daley-Yates and co-workers support this conclusion as they reported that differences displayed in PK studies between two salmeterol/fluticasone combination products (DPIs) could not be shown in a studies with pharmacodynamics end points.^(36,37)^ However, the studies might have had a limited sensitivity to show differences between the formulations as only one dose level was included. On the other hand, in a study where salmeterol chlorofuorocarbon (CFC) and non-CFC propellant MDIs were compared on 50, 150, and 300 μg doses the PK and pharmacodynamic result were in agreement.^(38)^ The higher systemic exposure, based on AUC_t_ and C_max_, following administration of CFC formulation, led to significantly greater systemic pharmacodynamic effects.

For budesonide/formoterol Easyhaler, the same batch was used in all four studies and it could be argued that the results are influenced by changes over time in the test batch rather than by different reference batches. That, however, was not the case. The time between the administration of the first and the last dose in the PK studies was approximately 14 months. During that time the test batch was analyzed four times and its FPD levels remained stable. This is in accordance what we have seen for both budesonide/formoterol Easyhaler and Symbicort Turbuhaler. FPDs do not change remarkably along the aging of the product batch.

The selection of the reference product batch to be used in the BE study is the responsibility of the sponsor and it is advisable to investigate several batches when selecting a reference product batch for the study.^(39)^ However, there are no criteria available for a representative reference batch. The number of batches studied before the first two studies was 6, before the last two studies 24, and by the end of the budesonide/formoterol Easyhaler registration process FPD results of altogether 28 reference batches were available. Obviously the choice of the reference batch for the first studies was not completely successful, as the FPDs of that batch were later found to be somewhat low. The studies conducted clearly show that the reference batch selection is crucial and can have a major impact on the results.

## Conclusions

Equivalence regarding both safety and efficacy between two OIPs, budesonide/formoterol Easyhaler and Symbicort Turbuhaler was shown based on totality of evidence from four PK studies and IVIVC analyses, and therefore, therapeutic equivalence between the products can be concluded. The results of the PK studies are likely dependent on the variability of FPDs of the reference product batches.
